# Review of Infant Feeding: Key Features of Breast Milk and Infant Formula

**DOI:** 10.3390/nu8050279

**Published:** 2016-05-11

**Authors:** Camilia R. Martin, Pei-Ra Ling, George L. Blackburn

**Affiliations:** 1 Department of Neonatology and Division of Translational Research, Beth Israel Deaconess Medical Center, Harvard Medical School, Boston, MA 02215, USA; cmartin1@bidmc.harvard.edu; 2 Department of Surgery, Feihe Nutrition Laboratory, Beth Israel Deaconess Medical Center, Harvard Medical School, Boston, MA 02215, USA; pling@bidmc.harvard.edu

**Keywords:** breast milk, infant formula, cow’s milk allergy, cow’s milk alternatives

## Abstract

Mothers’ own milk is the best source of nutrition for nearly all infants. Beyond somatic growth, breast milk as a biologic fluid has a variety of other benefits, including modulation of postnatal intestinal function, immune ontogeny, and brain development. Although breastfeeding is highly recommended, breastfeeding may not always be possible, suitable or solely adequate. Infant formula is an industrially produced substitute for infant consumption. Infant formula attempts to mimic the nutritional composition of breast milk as closely as possible, and is based on cow’s milk or soymilk. A number of alternatives to cow’s milk-based formula also exist. In this article, we review the nutritional information of breast milk and infant formulas for better understanding of the importance of breastfeeding and the uses of infant formula from birth to 12 months of age when a substitute form of nutrition is required.

## 1. Introduction

Mothers’ own milk is considered to be the best source of infant nutrition [1]. Extensive evidence has shown that breast milk contains a variety of bioactive agents that modify the function of the gastrointestinal tract and the immune system, as well as in brain development. Thus, breast milk is widely recognized as a biological fluid required for optimal infant growth and development. Recently, studies have further suggested that breast milk mitigates infant programming of late metabolic diseases, particularly protecting against obesity and type 2 diabetes [2].

The World Health Organization recommends that infants should be exclusively breastfed for the first six month of life [3]. The American Academy of Pediatrics also recommends breastfeeding for at least 12 months [4]. Recently, the Academy of Nutrition and Dietetics reaffirms and updates their mission that exclusive breastfeeding provides optimal nutrition and health protection for the first six months of life, and that breastfeeding with complementary foods from six months until at least 12 months of age is the ideal feeding pattern for infants [1]. In addition to its nutritional advantage, breastfeeding is convenient and inexpensive, and also is a bonding experience for the mother and infant.

The decision to breastfeed is highly personal and is often influenced by many factors [5]. Under certain situations, breastfeeding might not be possible, unsuitable or inadequate, which warrants an interruption or cessation in breastfeeding. Globally, only 38% of infants are exclusively breastfed. In the United States, only 75% of infants initiate breastfeeding from birth; however, by the age of three months, 67%, or 2.7 million, of them rely on infant formula for some portion of their nutrition [6]. Among new mothers, the six-month “any breastfeeding” rate for the total U.S. population is 43%, with only 13% meeting the recommendation to breastfeed exclusively for six months [4]. 

Infant formula is intended as an effective substitute for infant feeding [7,8]. Although production of an identical product to breast milk is not feasible, every effort has been taken to mimic the nutrition profile of human breast milk for normal infant growth and development. Cow milk or soymilk are most commonly used as the base, with supplemental ingredients added to better approximate the composition to human breast milk and to attain health benefits, including iron, nucleotides and compositions of fat blends. The fatty acids of arachidonic acid (AA) and docosahexenoic acid (DHA) are added. Probiotics and compounds, produced by genetic engineering, are either added or currently being considered for addition to formula. 

During the first six months of infant life, providing optimal nutrition is critical as the consequences of inadequate nutrition can be very severe. The purpose of this article is to review nutritional information on breast milk and infant formulas to reinforce the importance of breastfeeding, while also understanding the uses of infant formula. 

## 2. Human Breast Milk

Human breast milk contains carbohydrates, protein, fat, vitamins, minerals, digestive enzymes and hormones. In addition to these nutrients, it is rich in immune cells, including macrophages, stem cells, and numerous other bioactive molecules. Some of these bioactive molecules are protein-derived and lipid-derived, while others are protein-derived and indigestible, such as oligosaccharides. Human milk oligosaccharides (HMOs) possess anti-infective properties against pathogens in the infant gastrointestinal tract, such as *Salmonella,* *Listeria,* and *Campylobacter*, by flooding the infant gastrointestinal tract with decoys that bind the pathogens and keep them off the intestinal wall [9]. Oligosaccharides also play a vital role in the development of a diverse and balanced microbiota, essential for appropriate innate and adaptive immune responses, and help colonize up to 90% of the infant biome [10]. 

### 2.1. Composition of Human Breast Milk

Human breast milk is a complex matrix with a general composition of 87% water, 3.8% fat, 1.0% protein, and 7% lactose. The fat and lactose, respectively, provide 50% and 40% of the total energy of the milk [11]. However, the composition of human breast milk is dynamic and changes over time, adapting itself to the changing needs of the growing child. For instance, during each nursing session, the milk that is expressed first (foremilk) is thinner with a higher content of lactose, which satisfies a baby’s thirst, and following the foremilk, hindmilk, is creamier with a much higher content of fat for the baby’s needs. Variations are also present with the stage of nursing (age of infant), maternal diet, maternal health, and environmental exposure. During early lactation, the protein content in human milk ranges from 1.4–1.6 g/100 mL, to 0.8–1.0 g/100 mL after three to four months of lactation, to 0.7–0.8 g/100 mL after six months [11,12]. The fat content varies significantly with maternal diet and is also positively related to weight gain during pregnancy. Remarkably, it has been observed that a mother’s breast milk is almost always adequate in essential nutrients for her term infant’s growth and development, even when her own nutrition is inadequate. Although the mean concentrations of protein, sodium, chloride and potassium in early preterm milk are adequate to meet the estimated requirements for preterm infants, specific nutritional supplementation is required for mother’s milk delivered to preterm infants [13,14].

In contrast to protein and fat, lactose content is fairly constant in mature milk (after 21 days postpartum). The stable concentration of lactose is important in maintaining a constant osmotic pressure in human milk. Lactose also aids the absorption of minerals and calcium. In breast milk, many carbohydrate-based bioactive compounds, such oligosaccharides, are attached to lactose. If the small intestine does not produce enough of an enzyme (lactase) to digest these sugar complexes, lactose malabsorption and intolerance syndromes can be observed. Lactase deficiency malabsorption and disease are extremely rare in the exclusively breastfed infant. 

### 2.2. Protein in Human Breast Milk

There are two classes of protein in breast milk: Casein and whey. Casein becomes clots or curds in the stomach; while whey remains as a liquid and is easier to digest. Depending on the stage of milk, 80% to 50% of protein in breast milk is whey [11]. The whey/casein ratio in human milk fluctuates between 70/30 and 80/20 in early lactation and decreases to 50/50 in late lactation [15]. This proportion is significantly greater compared to the milk of other mammals. In cow’s milk, whey proteins represent only 18% of milk protein. Traditionally, infant formulas are high in casein, making them harder to digest compared to human breast milk. Because the amino acid profiles of casein and whey proteins are different, the overall amino acid profile of human milk varies depending on the stage of lactation. Glutamine, the most abundant free amino acid, is nearly 20 times higher in mature milk than its lowest value in colostrum [16]. Glutamine is important for providing ketoglutaric acid for the citric acid cycle, possibly acting as a neurotransmitter in the brain, and serving as a major energy substrate for intestinal cells [17].

The main whey proteins are alpha-lactalbumin, lactaferrin and secretory IgA. Other proteins include lysozyme, folate-binding protein, bifidus factor, casein, lipase and amylase, alpha1-antitrypsin and antichymotrypsin, and haptocorrin [11]. After ingestion, these proteins are broken down rapidly to free amino acids for absorption and utilization. Most of these proteins also have bioactive functions and non-nutritive functions [18]. For instance, alpha-lactalbumin is essential for lactose synthesis and binding of Ca and Zn ions. Casein assists to form masses with calcium and phosphorus. Lactoferrin and lysozyme prevent the spread of potentially pathogenic bacteria, preventing illnesses in infants. The IgA antibody destroys bacteria and protects the mucosal surface of the gut.

### 2.3. Fats in Human Breast Milk

Fats are the most important composition of breast milk, supplying energy and helping the development of the central nervous system. Moreover, milk fat is a carrier of taste and aroma. In general, human breast milk fat content ranges from 3.5% to 4.5% during lactation. The main lipid fraction are triglycerides, which account for about 95% of total lipids. Near half of milk fatty acids are saturated fatty acids, with 23% palmitic acid (C16:0) in total fatty acids [11]. The monounsaturated fatty acid, oleic acid (18:1w9), is in the highest percentage (36%) in milk. Human breast milk also contains two essential fatty acids, linoleic acid (C18:2w6) at 15% and alpha-linolenic acid (C18:3w3) at 0.35% [11]. These two essential fatty acids are, respectively, converted to arachidonic acid (AA, C20:4w6) and eicosapentaenoic acid (EPA, C20:5w3), the latter of which is further converted to docosahexaenoic acid (DHA, 22:6w3). AA, EPA and DHA are important for regulating growth, inflammatory responses, immune function, vision, cognitive development and motor systems in newborns.

Long chain polyunsaturated fatty acids are transferred from mother to fetus in the third trimester through the placenta, and to infants through breast milk after birth [19]. During the last trimester and neonatal period, brain tissue is rapidly synthesized. Cell differentiation and development of active synapses in the brain need specific requirements of DHA and AA. Eighty percent of brain DHA is acquired from the 26th week of gestation until birth. Notably, the synthesis of AA and DHA from linoleic acid (18:2w6) and alpha-linolenic acid (18:3w3) is limited in the fetus and neonate due to the premature enzyme activity. Thus, the required amounts of AA and DHA must come from the mother during pregnancy, or as breast milk after birth. One study has showed that the fat content and the percentage of all polyunsaturated fatty acids in breast milk increase significantly between the sixth week and sixth month of lactation [20]. There is evidence that slowly turning-over maternal body pools of AA are the major source of milk AA [21]. The AA concentration in breast milk is dose-dependently associated with the consumption of AA-rich foods in lactating mothers [22]. Breast milk EPA and DHA concentrations are also closely linked to maternal dietary EPA and DHA intake [23]. Human milk from lactating women consuming vegan or vegetarian diets has <0.1% DHA, compared to mean levels of 0.2%–0.4% DHA in the United States and ≥0.8% DHA in China, where DHA intakes from fish or other sources are high [24]. It is suggested that intakes of ~300 mg of DHA per day are necessary to achieve human milk levels of 0.3%–0.35% of DHA [25]. However, the effects of human milk fatty acids on neurodevelopment is complex, particularly because neurodevelopment is assessed after the period of the first six month of exclusive human milk feeding.

In premature birth, the transmission of these fatty acids is interrupted from the placenta to the fetus during the critical last trimester. Studies also showed that decreased postnatal docosahexaenoic and arachidonic acid blood levels in premature infants are associated with neonatal morbidities [26]. Thus, after birth, the preterm infant is dependent on an adequate diet for sufficient fatty acid levels. Adding DHA and AA to preterm-infant formulas led to initial beneficial effects on visual acuity, visual attention and cognitive development compared with infant receiving no supplementation [27].

### 2.4. Vitamins, Minerals and Other Bioactive Components in Breast Milk

Human breast milk contains adequate amounts of most vitamins to support normal infant growth, except for vitamins D and K. Infants who are exclusively breastfeeding receive below the minimum recommended intake of vitamin D, and much lower than the recommended dietary intake. These infants are at the risk for vitamin D deficiency, inadequate bone mineralization and conditions such as rickets. However, the overall risk of vitamin D deficiency in breastfed infants is also correlated with overall sun exposure with increasing risk in climates with a lower sun index. Maternal supplementation with 400–2000 IU (International Unit). of vitamin D/day can increase the levels of vitamin D in breast milk, but only a higher dose (2000 IU) achieves satisfactory levels of 25-OH-D in the infant [11]. Normal vitamin D stores present at birth are depleted within eight weeks. Sunlight exposure and vitamin D supplementation are recommended for breastfed infant. Formula-fed infants often have higher serum concentration of vitamin D metabolites than breastfed-infants. Vitamin K is essential to the protein involved in blood coagulation. However, only limited amounts of vitamin K is transferred from the placenta to fetus. Thus, a newborn infant often has an extremely low concentration of vitamin K, and is at risk of developing hemorrhagic disease. After birth, vitamin K supplementation is recommended.

In human breast milk, minerals contribute to a variety of physiological functions, forming essential parts of many enzymes and are of biological important to molecules and structures. The contents of minerals are comparable between human milk and bovine milk. Over the decades, many other bioactive components have been identified in human milk, including hormones, growth factors and immunological factors. 

## 3. Human Milk Options—Milk Donors and Milk Banks

The World Health Organization and the American Academy of Pediatrics recommends pasteurized human donor milk for preterm infants when a mother’s own milk is unavailable [28]. Donor milk undergoes a pasteurization process, which reduces many of the normal commensal microbes, as well as significantly reducing or obliterating live immune cells, bioactive proteins, and enzymes, collectively limiting some of the health benefit compared to a mother’s own milk. Research efforts to optimize donor breast milk are ongoing. Before using donor breast milk, the mother should consult with their baby’s health care providers.

## 4. Infant Formulas

Infant formula is intended as an effective substitute to breast milk and is formulated to mimic the nutritional composition of breast milk. The recently updated FDA (Food and Drug Administration) rule on current Good Manufacturing Practices for infant formula, 21 CFR 106.96 [6], requires, among other things, that formulas satisfy the quality factors of normal physical growth and a sufficient biological quality of protein component (adequate amounts of protein in a form that can be used by infants). Infant formula is only for the health of infants without unusual medical or dietary problems. The manufacturing process is highly regulated and monitored to meet national and international quality criteria [29,30].

### 4.1. The Infant Formula Market

The United Nations estimates that the world’s current population of 7.2 billion will grow by one billion over the next 12 years, reaching 9.6 billion by 2050 [31]. This increase will drive the global demand for infant milk formula, especially innovative products that use ingredients such as prebiotics and specific milk protein fractions [31]. Over the next five years, the $50 billion infant formula market is expected to be the fastest-growing packaged food category, achieving gains in excess of 7% a year [32]. Some industry experts predict an even higher annual growth of 8%–9% [32]. According to analyst Diana Cowland, the rapid expansion of infant formulas is set to continue with a compounded annual growth rate of 11%, with demand driven by Asia, and more particularly, China [33]. 

Infant formulas are available in three forms: (1) powder: The least expensive form of infant formula that must be mixed with water before feeding; (2) liquid: Concentrated liquid that must be mixed with an equal amount of water; and (3) ready-to-feed: The most expensive form of infant formula that requires no mixing.

### 4.2. Guidelines for Manufacturing of Infant Formula

Infant formulas must include proper amounts of water, carbohydrate, protein, fat, vitamins and minerals. The composition of infant formula is strictly regulated, and each manufacturer must follow established guidelines set by government agencies. For instance, all the major components added to formula (protein, lipids, carbohydrates) have a range of minimum and maximum values for their effectiveness. These components must have established a history of safe use [34]. The required range of each nutrient must be maintained throughout the shelf life of the product [35]. For amino acids, only L forms of amino acid are allowed to be added, while D forms are not permitted because they may cause D-lactic acidosis [36]. Fructose should be avoided due to fructose intolerance. Hydrogenated fats and oils are also not allowed. Ionizing radiation of the formula product is not permitted because it could cause product deterioration [35]. Infant formula prepared ready for consumption should contain no less than 60 kcal (250 kJ) and no more than 70 kcal (295 kJ) of energy per 100 mL (CAC, 1981) [11]. Furthermore, product reformulation must be based on medical and nutritional findings. The committee of the “Evaluation of the addition of Ingredients New to Infant Formula” has recommended that “manufacturers must demonstrate that the formula containing the new ingredient is capable of sustaining physical growth and development over 120 days when formula is likely to be the sole source of infant nutrition” [7]. 

In the United States, the Food and Drug Administration (FDA) defines that adding new ingredients to infant formula should have “reasonable certainty of no harm” as the safety standard [7]. The World Health Organization (WHO) has noted that unmodified cow’s milk should never be fed to infants, and that unmodified goat’s milk is also not recommended for infants. With the WHO guidelines, federal and local agencies of different countries control and monitor infant formula regulations, including requirements for quality and manufacturing practices in their own countries. From a manufacturers perspective, it is in their best interest to continuously improve their products to be as close as possible to human breast milk.

### 4.3. Classes of Infant Formula Products

There are three major classes of infant formulas: Cow-milk based formula, soy-based formula and specialized formula. They vary in nutrition, calories, taste, digestion, and cost. Specific kinds of formulas are available to meet a variety of needs. Some cow’s milk substitutes are amino acid based or contain extensively hydrolyzed whey or casein proteins. Some are rice-based formula.

### 4.4. Cow Milk-Based Formula

Bovine milk is the basis for most infant formula. However, bovine milk contains higher levels of fat, minerals and protein compared to human breast milk. Therefore, cow milk must be skimmed and diluted to more closely resemble human breast milk composition [34,35]. Cow-milk-based infant formula contains added vegetable oils, vitamins, minerals and iron for consumption by most healthy full term infants.

According to the American Academy of Pediatrics [37], children under one year of age should not be fed raw, unmodified, or unpasteurized cow’s milk as a replacement for human milk or infant formula. Additionally, unmodified milk does not provide enough vitamin E, iron or essential fatty acids. Moreover, infants’ systems cannot handle the high levels of protein, sodium, and potassium of unmodified cow milk. Formulas with a protein content 2–2.5 g/100 mL and a protein/energy ratio <3 g/100 kcal are used for normal infants, while with higher protein content (2.9 g/100 mL) and higher protein/energy ratio (3.5 g/100 kcal) are for a very low birth weight or preterm infants [38]. Recent studies showed that high protein content in infant formula is associated with excess weight gain in infancy, which can lead to a 20% risk of obesity later in life [39]. 

Cow’s milk is one of the first foods introduced into an infant’s diet and one of the most common causes of food allergy [40]. Usually, clinical reactions start very early in life, after breastfeeding has stopped and cow’s milk is introduced into the diet; symptoms rarely appear during lactation. 

The clinical manifestations of cow milk allergy vary widely in type and severity. It may be defined as a reproducible adverse reaction to one or more milk proteins (usually caseins or whey beta-lactoglobulin) mediated by at least one immune mechanism [41]. The prevalence of cow milk allergy varies across studies, as well as across diagnostic criteria and infant diets. It presents in the first year of life, with an estimated population prevalence of between 2% and 3% [41], or as high as 7% [42]. However, the results from recent cohort studies and from a randomized trial of early introduction of allergenic foods in the diet of breast-fed infant have shown that the incidence for IgE-mediated cow milk allergy could be as low as 0.5% [43,44,45]. Because there is no definition for differentiating IgE-mediated and non-lgE-mediated cow milk allergy, and the clinical symptoms of both overlap significantly, it is possible that, at least, some non-IgE mediated allergy cases may have been included in IgE-mediated cow milk allergy in previous reports. 

Symptoms of cow milk allergy may be immediate or delayed. IgE-associated mechanisms are responsible for approximately 60% of cow milk-induced adverse reactions. These typically appear immediately or within 1 to 2 h after ingestion, and tend to affect the skin, respiratory system, and gastrointestinal tract. In severe cases, cow milk allergy can also cause systemic anaphylactic reactions [40]. 

Non-IgE-associated symptoms are characterized by delayed onset of approximately 2 h to several days after cow milk consumption. The period of 2 h helps to exclude the non-IgE-associated or no-allergic reactions. The non-lgE-associated clinical symptoms mainly affect the gastrointestinal system, and include enterocolitis, proctocolitis, enteropathy, and eosinophilic esophagitis [46]. Non-lgE-mediated gastrointestinal food-induced allergic disorders have a favorable prognosis and majority symptoms dissolve within one to five years [47].

### 4.5. Soy-Based Formulas

Formulas made from soy proteins are effective options for infants with galactosemia or congenital lactase deficiency. They help with colic and milk allergies, however, rarely, infants who are allergic to cow’s milk may also be allergic to soymilk [33]. Soy products should not be used in infants under six months of age with food allergy [46]. Because phytoestrogens are present in soy-based formula, the uses of soy-based formulas are limited by the concern of potential harm for the infant, although this remains controversial [48,49].

### 4.6. Hypoallergenic Formulas

Protein hydrolysate formulas are meant for infants and babies who are unable to tolerate cow milk or soy-based formulas. They contain protein that has been hydrolyzed—partially or extensively—into smaller sizes than those found in cow or soy-based products. For infants who have a protein allergy, extensively hydrolyzed formulas are a satisfactory alternative. 

### 4.7. Amino Acid Formulas

Amino acid formulas are another option for infants who have severe cow milk allergy with reactions to or refusal to ingest appropriate amounts of extensively hydrolyzed formula. They provide protein in the form of free amino acids with no peptides.

## 5. Non-Bovine Milk Sources

Elimination of all cow milk products without appropriately modified and fortified substitutions can lead to malnutrition and/or specific nutrient deficiencies at a time when infants and children are growing. Infant milk formulas from different animals (goat, ewe, mare, donkey, or camel), or formulas based on lamb or chicken, have been widely marketed as substitutes for cow milk in the management of cow milk allergy in infants and children. However, other animal-milk-based formulas are currently not acceptable in many places because there are no robust randomized clinical trials.

## 6. Probiotics and Prebiotics

The high concentration and structural diversity of human milk oligaosaccharides are unique to humans. Without probiotics and prebiotics supplementation, the gut microbiota of formula-fed infants is generally not dominated by the Bifidobacterium species [50,51]. Studies have shown that breastfed newborn carry a more stable and uniform population of oligaosaccharides compared with formula-fed newborns [52]. Adding probiotics to formula represents a key strategy to reduce the incidence and severity of diarrhea in infants [53].

Domestic animal milk contains a large variety of complex oligosaccharides. Sialylated oligosaccharides account for approximately 80%–90% of the total pool from milk of all domestic animals [54]. Milk of grazing cows contains higher concentrations of sialic acid compared to non-grazing cows [55]. Cow’s milk might be a useful source of a variety of sialylated oligosaccharides for use as additives in infant formula. It is now also possible to supplement commercial infant formula with synthesized oligosaccharides that are chemically identical to human milk oligosaccharides [56].

Most probiotic strains added to formula have been isolated from food or fecal infant microbiota. Although the use of probiotics is now extending from research to recommendations, rigorous scientific effort is still required to validate specific strains with antiallergenic potential for preventive and therapeutic applications. 

Evidence on the use of *Lactobacillus reuteri* for the treatment of infantile colic is mixed. Sung *et al.* [57] found that *L. reuteri* DSM 17938 was of no benefit in a community sample of breast- and formula-fed infants with colic. This outcome differed from those of smaller trials in select populations, but did not support a general recommendation for the use of probiotics to treat colic. Conversely, a prospective, randomized, blinded, controlled trial in 138 infants showed that *L. reuteri* had a protective effect. The treatment group showed a lower number of pediatric consultations related to infant colic than the control group (*p* < 0.0001). It also reduced the use of pain relieving agents and of infant formula [58]. Such new treatments as probiotics have been proposed to treat infantile colic, but only a few strains have been tested. Further investigations are needed to provide evidence-based guidelines.

The impact of early pre- and probiotic intervention on preterm infants’ well-being, crying, growth, and microbiological programming were conduced in 94 infants (gestational age 32 to 36 weeks and birth weight >1500 g) who were randomized to receive prebiotics (a mixture of galacto-oligosaccharide and polydextrose 1:1), probiotics (*Lactobacillus rhamnosus* GG), or placebo during the first two months of life and follow-up lasted one year [59]. The results showed that among excessive criers (29% of the infants), there was significantly less crying in the pre- and probiotic groups than in the placebo group (19% *vs.* 19% *vs.* 47%, respectively; *p* = 0.02). The placebo group had a higher percentage of *Clostridium histolyticum* bacteria in their stools than the probiotic group did (13.9% *vs.* 8.9%, respectively; *p* = 0.05). Another study on safe and simple strategies to prevent viral respiratory tract infections between 3 and 60 days of life has demonstrated that prebiotics or probiotics had significantly less (*p* < 0.001 and *p* = 0.022) viral respiratory tract infections compared with those receiving placebo [60]. Additionally, the incidence of rhinovirus-induced episodes, which comprised 80% of all respiratory tract infections, was significantly lower in the prebiotic (*p* = 0.003) and probiotic (*p* = 0.051) groups than in the placebo group.

## 7. Fatty Acids and Milk Fats from Different Mammalian Species

The lipid portion of human milk is the major source of energy for growing infants and provides approximately 45% to 55% of total energy. The lipid compositions of mammalian milks (cow, buffalo, donkey, sheep, and camel) were compared with that of human milk on fatty acid profiles and triacylglycerol (TAG), phospholipid, and phospholipid fatty acid compositions, as well as melting and crystallization profiles [59]. The results showed that these milk fats, especially sheep milk fat, had high degrees of similarity to human milk fat in total fatty acid composition. However, other chemical aspects had less similarity. This outcome indicates that these milk fats do not meet the requirements of human milk fat substitutes, but large amounts of these commercialized mammalian milk fats are good raw materials for infant formula production. Milk fat globule membranes are a fraction that has been previously excluded from infant formulas, but its components are active and prevent infection [61]. Milk fat globule membrane supplementation of infant formula also narrows the gap in cognitive development between breastfed and formula fed infants [62]. 

## 8. Bioactive Proteins

Novel dairy fractions from bovine milk have been isolated and are now commercially available. Many of these components are proteins, such as α-lactalbumin, lactoferrin, osteopontin, and milk fat globule membrane proteins. When adding bioactive proteins to infant formulas, it is important to reduce the total protein content of formula. The amino acid composition of formula is also important; serum concentrations of essential amino acids should not be lower than those in breastfed infants. For example, α-Lactalbumin, often the first limiting amino acid in infant formulas, is digested into smaller peptides with antimicrobial and prebiotic activities and has an immunostimulatory effect. It also enhances mineral absorption. Osteopontin is a heavily phosphorylated and glycosylated protein that modulates immune function and stimulates Th1/Th2 switching. It might also affect bone mineralization and growth, and facilitate the biological function of lactoferrin. 

## 9. Conclusions

Breast milk is the best nutrition for infant growth and development, and is also rich in antibodies that provide the first source of adaptive immunity in a newborn’s intestinal tract. In preterm or low birth weight newborns, a mother’s own milk is the first choice for preterm infants; when it is unavailable, donor breast milk is considered as the next best choice. For healthy newborns whose mothers are unable to provide sufficient breast milk, the current option of choice is infant formula. 
